# Fluoroquinolone resistance in *Campylobacter jejuni* and *Campylobacter coli* from poultry and human samples assessed by PCR-restriction fragment length polymorphism assay

**DOI:** 10.1371/journal.pone.0199974

**Published:** 2018-07-06

**Authors:** Yuli Melisa Sierra-Arguello, Thales Quedi Furian, Gustavo Perdoncini, Hamilton L. S. Moraes, Carlos T. P. Salle, Laura B. Rodrigues, Luciana Ruschel dos Santos, Marcos José Pereira Gomes, Vladimir Pinheiro do Nascimento

**Affiliations:** 1 Center for Diagnosis and Research on Avian Pathology (CDPA), Faculty of Veterinary Medicine, Federal University of Rio Grande do Sul (UFRGS), Porto Alegre, Brazil; 2 Laboratory of Veterinary Bacteriology, Faculty of Veterinary Medicine, Federal University of Rio Grande do Sul (UFRGS), Porto Alegre, Brazil; 3 Faculty of Veterinary Medicine, University of Passo Fundo (UPF), Passo Fundo, Brazil; Australian National University, AUSTRALIA

## Abstract

The objective of this study was to determine fluoroquinolone resistance in *Campylobacter* spp from poultry and human isolates. Forty-one *Campylobacter jejuni* isolates (30 of poultry origin and 11 of human origin) and 11 *Campylobacter coli* isolates (10 of human origin and 1 of poultry origin) were examined for ciprofloxacin, norfloxacin, and nalidixic acid resistance using the minimal inhibitory concentration (MIC) method. Thereafter, the isolates were analyzed by PCR–Restriction Fragment Length Polymorphism (RFLP) assay for detection of Thr-86 mutation. Finally, DNA sequencing was performed for confirmation of *gyr*A gene mutation. A complete correlation was observed between MICs, PCR-RFLP assay, and sequencing. The results revealed high quinolone resistance rates for *C*. *jejuni* (100%) and *C*. *coli* (100%) isolates obtained from poultry and moderate resistance for *C*. *jejuni* (9.1%) and *C*. *coli* (40%) samples of human origin. A mutation in codon 86 of the *gyr*A gene with a Thr-to-Ile substitution is reported to be the main cause of high resistance to quinolones. This mutation can be analyzed by PCR-RFLP assay, which has been proven to be a simple and fast method for the detection of fluoroquinolone resistance in *Campylobacter* spp.

## Introduction

Campylobacteriosis is one of the most important bacterial foodborne diseases in humans worldwide and, therefore, a major public health concern. Despite the importance of *Campylobacter* spp detection in poultry meat and in its by-products, relatively few studies exist on the occurrence, epidemiology, and antimicrobial resistance of this pathogen. The poultry industry is one of the most important sectors in the Brazilian economy–chicken meat exports have ranked first since 2004, and Brazil is the third world producer of chicken meat, outranked only by the United States, as pointed out by the 2017 Annual Report on Brazilian Poultry [1]. Therefore, an overview of the studies on the occurrence of *Campylobacter* in the Brazilian chicken processing chain is of paramount importance. Two thermotolerant species–*Campylobacter jejuni* (*C*. *jejuni*) and *Campylobacter coli* (*C*. *coli*)–are responsible for the vast majority of human infections, among which 80–90% are caused by *C*. *jejuni* [2], [3]. This bacterium is transmitted to humans by contaminated foods of animal origin, especially undercooked poultry meat and unpasteurized milk/dairy products [4]. *Campylobacter* species can cause gastrointestinal and systemic infections. Serious long-term sequelae of these infections in humans include Guillain-Barré syndrome, Miller Fisher syndrome, reactive arthritis, Reiterʼs syndrome, hemolytic uremic syndrome, and septicemia [5–10].

Antimicrobial treatment is indicated when patients suffer from recurrent or systemic *Campylobacter* infection. Macrolides and fluoroquinolones are used to treat *Campylobacter* infections in humans [11], [12]. As fluoroquinolones play an important role in the clinical treatment of human campylobacteriosis, antimicrobial resistance of *C*. *jejuni* and *C*. *coli* strains has become a public health concern. Fluoroquinolone resistance is primarily associated with a single threonine at position 86 to isoleucine (Thr-86-to-Ile) mutation in *gyr*A gene in isolates from humans and animals [13–15]. The purpose of the present work was to determine fluoroquinolone resistance of *Campylobacter* isolated from poultry slaughterhouses and human subjects in Brazil. The antimicrobial resistance of *Campylobacter* spp. strains was determined using the broth microdilution test and the underlying mechanism of resistance was analyzed using molecular methods: a PCR-based restriction fragment length polymorphism (PCR–RFLP) analysis and sequencing for confirmation of *gyr*A gene mutation.

## Materials and methods

### Bacterial strains and growth conditions

This study was carried out in three federally inspected slaughterhouses in southern Brazil between January and December 2012, where 60 samples were analyzed for the presence of *Campylobacter* spp. A total of 31 *Campylobacter* strains were isolated and identified from poultry (carcasses: n = 30; chiller water samples: n = 1). Additionally, *Campylobacter* strains isolated from human patients with gastroenteritis were randomly obtained. Human clinical isolates were obtained from the Culture Collection of the Oswaldo Cruz Institute (IOC) in Rio de Janeiro, Brazil (n = 21) and the swabs were stored in transport medium at 0–4°C for no more than 48 h before microbiological examination (Transystem Amies medium transport swabs; 108.USE, Copan Diagnostics Inc, Murietta, USA). Of these, 11 isolates had been previously identified as *C*. *jejuni* and 10 as *C*. *coli*, confirmed by molecular analysis.

#### Sampling of broiler carcasses

Fresh disposable gloves were worn to remove each carcass from the processing line. Each carcass was placed in a sterile plastic bag, and carcasses were transported to the laboratory in insulated boxes with ice packs. Immediately upon arrival at the laboratory, rinse samples were collected by shaking carcasses for 1 min after the addition of 400 mL of buffered peptone water (BPW 1%) (CM1049 Oxoid®). After shaking, 1 mL of each sample was immediately transferred to 9 mL of Bolton broth (CM0983 Oxoid®, supplement SR0183) and incubated at 41.5°C under microaerophilic conditions (5% O_2_, 10% CO_2_, and 85% N_2_) for 48 h.

Isolation was performed in accordance with the International Organization for Standardization guidelines [16]. Thereafter, 10 μL was streaked onto a modified charcoal cefoperazone deoxycholate agar (mCCDA) plate (CM739, Oxoid®, with cefoperazone selective supplement SR 155E) and incubated at 41.5°C for 48 h under microaerophilic conditions. Presumptive *Campylobacter* colonies were cultivated on blood agar plates (BA; Blood Agar Base N.2, Oxoid®, supplemented with 5% sterile defibrinated sheep blood) and incubated for 48 h under the above-mentioned conditions. *Campylobacter* species from the primary culture were initially identified by colony appearance, Gram staining, growth in oxygen, and oxidase test. The colonies were collected and suspended in 1 mL of ultrapure water, transferred to microtubes, and later frozen at -20°C until DNA extraction. All isolated strains were stored in cryovials with brain heart infusion broth (BHI, Becton Dickinson, Sparks, MD, USA) and 20% (1 Vol/1Vol) glycerine at -80°C.

#### Sampling and examination of chiller water

Chiller water (100 mL) was sampled by immersing sterile plastic containers in it. The water was transported to the laboratory in an insulated container with ice packs or in a portable refrigerator for enrichment and enumeration of the *Campylobacter* spp as described above.

### DNA extraction and species confirmation by PCR

Genomic DNA was extracted using a modified protocol described by Borsoi *et al*. [17]. Briefly, isolated colonies were picked from BA plates and suspended in 1 mL of distilled water in a microcentrifuge tube. Samples were heated for 10 min at 95°C before being added to the PCR mix with specific primers selected from *map*A and *ceu*E genes for simultaneous detection of *C*. *jejuni* and *C*. *coli*, respectively. All isolates were identified by mPCR according to a method previously developed by Denis *et al*. [18] and Linton *et al*. [19] with some modifications.

### Minimal inhibitory concentration (MIC)

The minimal inhibitory concentrations (MICs) of ciprofloxacin, norfloxacin, and nalidixic acid in all isolates were determined by the broth microdilution method according to the Clinical and Laboratory Standards Institute (CLSI) guidelines [20], [21]. The use of MIC has been advocated by some authors for treatment of serious infections, septicemia, or for treatment of immunosuppressed patients. The antimicrobials were tested in a twofold dilution series: ciprofloxacin (Sigma, St Louis, MO, USA) 0.125–64 μg/mL, norfloxacin (Sigma) 0.125–64 μg/mL, and nalidixic acid (Sigma) 0.25–128 μg/mL. The microtiter plates were incubated for 24 h at 41.5°C under microaerophilic conditions. The MIC breakpoints for antimicrobial resistance were those recommended by the CLSI for non-*Enterobacteriaceae* [20], [21] concerning fluoroquinolones for which such recommendations are available: ciprofloxacin (MIC ≥4 mg/L), norfloxacin (MIC ≥16 mg/L), and nalidixic acid (MIC ≥32 mg/L). The organisms prescribed as reference strains for quality control procedures included those obtained from the American Type Culture Collection (ATCC), *Escherichia coli* ATCC 25922, *Staphylococcus aureus* ATCC 29213, *Pseudomonas aeruginosa* ATCC 27853, and *Campylobacter jejuni* ATCC 33560.

### Analysis of the quinolone resistance-determining region (QRDR) of *gyr*A

The resistance of *C*. *jejuni* and *C*. *coli* to quinolones depends mainly on mutations in the QRDR of the *gyr*A gene and was identified by PCR-RFLP. A PCR-RFLP assay using RsaI, common restriction enzyme, was used to identify a point mutation at Thr-86 in the gyr*A* gene product, involving the replacement of Thr-86 with Ile. The analysis of the *gyr*A gene mutation started with the amplification of a 179-bp fragment.

The PCR conditions were adapted [13], [22]. The PCR was carried out in 25 μL of reaction mixture containing 2.5 μL of 10X PCR buffer [200 mM Tris-HCl (pH 8.4), 500 mM KCl], 0.5 μL (5U/μL) of *Taq* thermostable DNA polymerase (Invitrogen®), 1 μmol 1^-l^ of MgCl_2_ (25 mM), 2 μL dNTPs (dATP, dCTP, dGTP, and dTTP, each at 2.5 mM), 1 μL of extracted template DNA, and 1 μL (10 pmol 1^-l^) of each primer. Sterile Milli-Q water was added q.s.p 25 μL. The sequences of primers and PCR conditions are listed in Table 1. All amplification reactions were performed in a thermal cycler (Peltier Thermal Cycler Biocycler–MJ96+/MJ96G). For visualization of PCR products, 10-μL aliquots were electrophoresed on 1.5% agarose gel (Invitrogen UltrapureTM Agarose®–Carlsbad, USA), stained with ethidium bromide, and the amplified products were visualized in a UV transilluminator (Pharmacia LKB Macro-Vue®). Amplification products of the expected size (179 bp) were obtained for all strains, whether they had been resistant or susceptible to ciprofloxacin.

**Table 1:** List of primers and PCR conditions used in this study.

Target gene	Primers	Sequence (5´- 3´)	PCR Conditions	Product (bp)	Reference
16S	MD16S1	ATCTAATGGCTTAACCATTAAAC	95°C/10 min, 35 cycles: 95°C/30s, 59°C/90s, 72°C/1 min, and 72°C/10 min.	857 for *Campylobacter* genus identification.	(18, 19)
rRNA	MD16S2	GGACGGTAACTAGTTTAGTATT			
*map*A	MDmapA1	CTATTTTATTTTTGAGTGCTTGTG		589 for *C*. *jejuni* species identification.	(18)
	MDmapA2	GCTTTATTTGCCATTTGTTTTATTA			
*ceu*E	col3	AATTGAAAATTGCTCCAACTATG		462 for *C*. *coli* species identification.	(18)
	MDcol2	TGATTTTATTATTTGTAGCAGCG			
PCR-RFLP (*gyr*A) *C*. *jejuni*	cjgyrAM1	AAATCAGCCCGTATAGTGGGTGCTGTTATAGGTCGTTATCACCCACACATGGAGGT	94°C/5 min, 30 cycles: 94°C/1 min, 51°C/1 min,72°C/45s, and 72°C/7 min.	179 detection *gyr*A *(C*. *jejuni)*.	(15, 22)
	cjgyrA2	TCAGTATAACGCATCGCAGC			
PCR-RFLP (*gyr*A) *C*. *coli*	colgyrA	AAATCTGCTCGTATAGTAGGGGATGTTATCGGTAAGTATCATCCACATGGCGGT	94°C/5 min, 30 cycles: 94°C/1 min, 55°C/1 min,72°C/45s, and 72°C/7 min.	179 detection *gyr*A *(C*. *coli)*.	(13, 15)
	colgyrA2	TCAGTATAACGCATCGCAGC			

Finally, the PCR products were digested with RsaI (PROMEGA®) to detect mutations at position Thr-86. Enzyme digestion was performed in a 20-μL mixture containing 2 μL of the PCR product and 1 μL of enzyme (10U/μL) following the manufacturer’s instructions. The amplified PCR products were digested with RsaI enzyme resulting in 125-bp and 54-bp fragments. The DNA segments were separated using 3% agarose gel (Invitrogen®). DNA bands were stained with ethidium bromide for 2 h at 100 V and viewed under UV light.

### DNA sequencing for *gyr*A gene mutation

The PCR products were purified using the QIAquick PCR purification kit (Qiagen, Valencia, CA, USA) for use with sequencing reactions. Both strands were sequenced with a reaction containing 80 ng of target DNA and five pmol of forward and reverse primers. Product sequences were analyzed on an automatic sequencer (ABI.PRISM 3100 Genetic Analyzer, Applied Biosystems, CA, USA). The resulting sequences were assembled and analyzed using the BioEdit Sequence Alignment Editor Software (version 7.0.9.0).

#### Accession numbers

The complete sequences of *C*. *coli* and *C*. *jejuni* of the *gyr*A genes can be found under GenBank accession numbers AF092101 and L04566, respectively.

#### Analysis of results

Resistance data were analyzed using the WHONET software, version 5.4. SPSS (version 18) was used for the statistical analysis. The chi-square test was chosen as statistical method.

## Results

**Fig 1:**
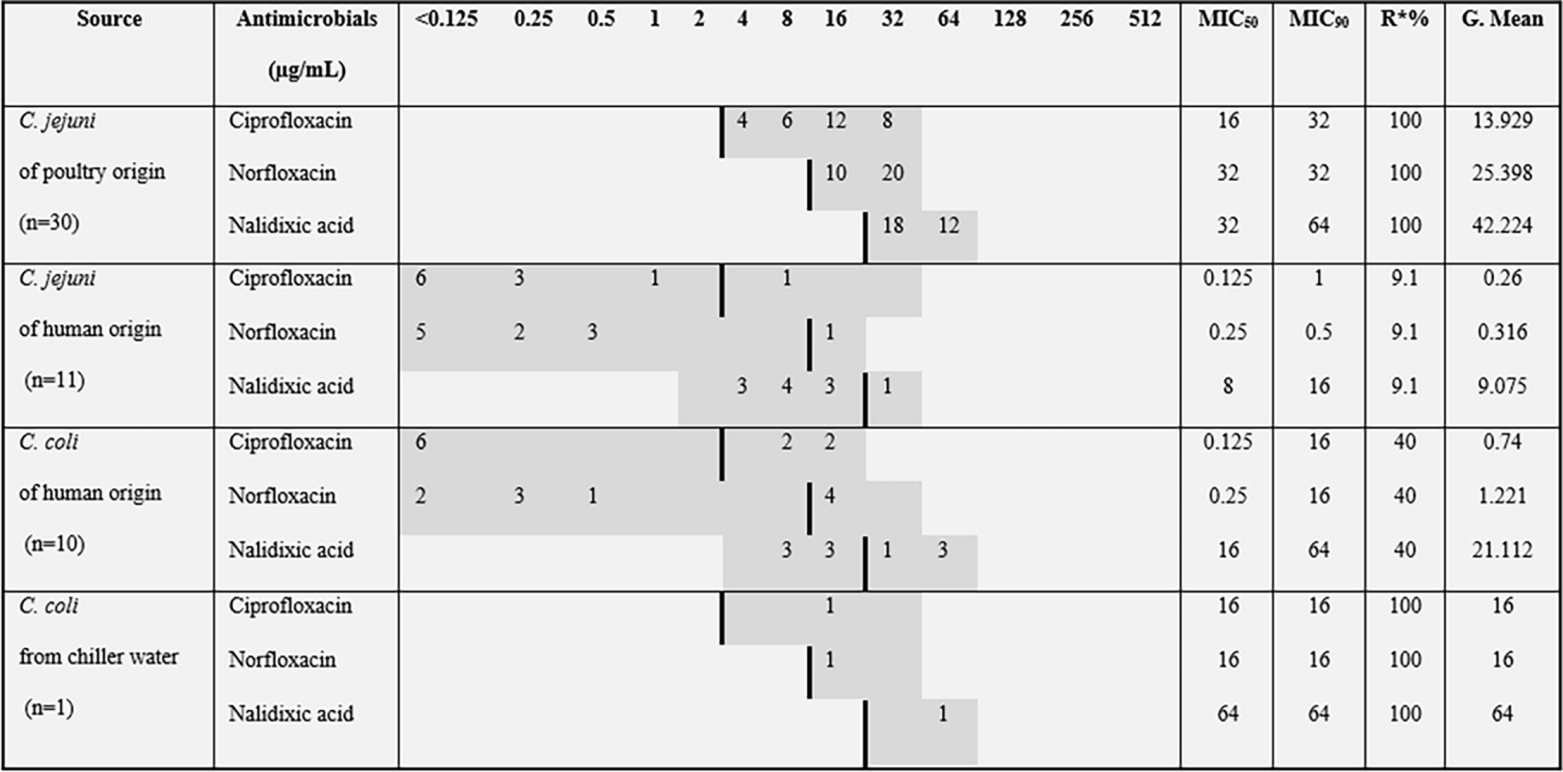
Distribution of MICs for *Campylobacter* spp. isolated from poultry and human samples. Breakpoint values, MIC values, and resistance rate of 52 *Campylobacter* strains. A thick black line indicates the breakpoint between clinically sensitive and resistant strains. Gray shadowed area indicates the test range (μg/mL) of each antimicrobial agent. MIC_50_ = (*n* χ 0.5); MIC_90_ = (*n* χ 0.9); R* = Resistance rate.

All 52 *Campylobacter* isolates were confirmed as *C*. *jejuni* (n = 41) and *C*. *coli* (n = 11) by mPCR. Antimicrobial susceptibility and resistance to each antimicrobial agent were calculated. MIC_50_ and MIC_90_ values, as well as rates of resistance, are displayed in Fig 1 (S1 and S2 Tables).

The resistance rate for *C*. *coli* and *C*. *jejuni* varied according to the source. One hundred percent of the isolates from poultry slaughterhouses were resistant to fluoroquinolones. By contrast, the sensitivity of human isolates of *C*. *jejuni* and *C*. *coli* to fluoroquinolones was 89% and 60%, respectively.

**Fig 2:**
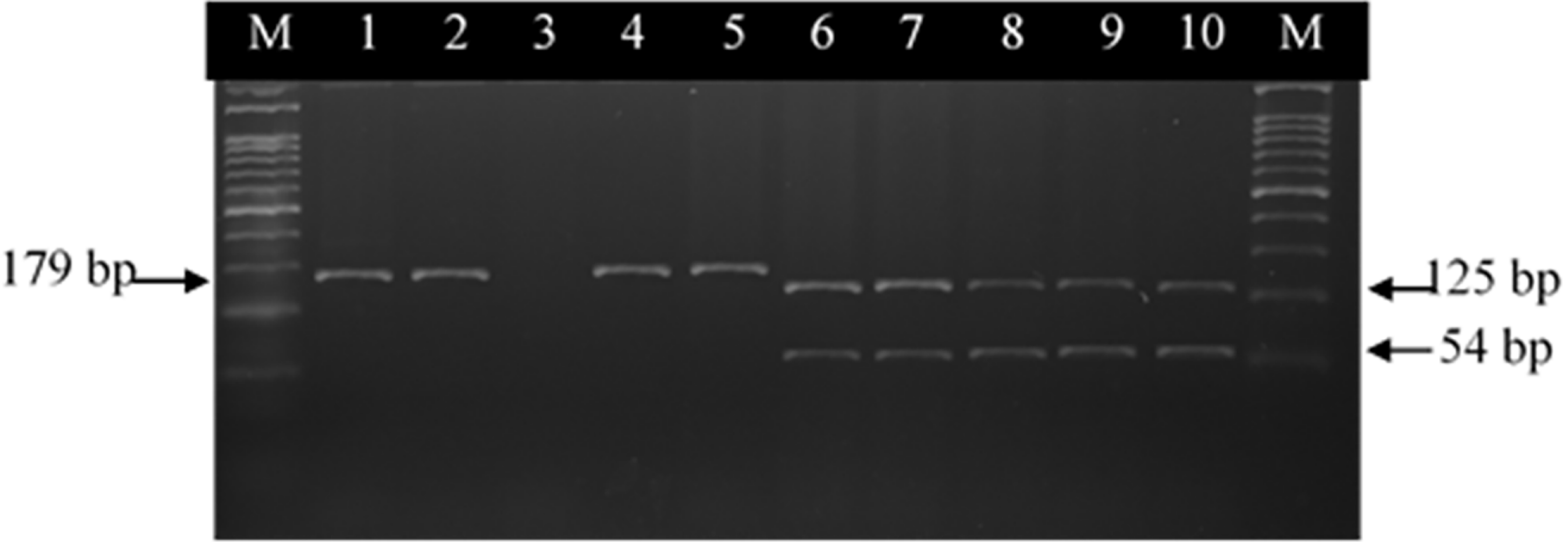
PCR-restriction fragment length polymorphism patterns obtained after digestion with RsaI in 10 *C*. *coli* strains. Lanes: M = 100-bp DNA Ladder (Invitrogen®); 1 to 2, undigested PCR product of *gyr*A gene; 3, negative control; 4 to 5, ciprofloxacin resistance; 6 to 10, ciprofloxacin-sensitive strains.

A close correlation was observed between PCR-RFLP and MICs. The analysis of restriction patterns after digestion with RsaI showed that all resistant strains had the same RFLP, the 179-bp fragment. These strains were assumed to have mutation at Thr-86. The susceptible strains had two fragments (54 bp and 125 bp) produced by RsaI digestion. These samples were assumed to have no mutation at Thr-86 (Fig 2, lanes 1,2,4,5). PCR products were sequenced, confirming the RFLP results. The highest rates of resistance of *Campylobacter* spp. were among poultry samples (100%).

## Discussion

Adaptation of microorganisms is characterized by their inherent capacity to mutate, evolve, and evade the stress response, allowing them to survive otherwise lethal environments [23]. The selection pressure obtained by the use of antimicrobials results in the development of resistance, either acquired or intrinsic, by modification of a host gene target [24].

In the present study, the isolates showed varying degrees of resistance to fluoroquinolone, depending on their origin. In general, *C*. *jejuni* and *C*. *coli* isolated from poultry showed high fluoroquinolone resistance (100%) whereas *C*. *jejuni* and *C*. *coli* from human samples had moderate resistance (9.1 and 40%, respectively). Resistance to fluoroquinolones in human samples was found more often in *C*. *coli* (40%) than in *C*. *jejuni* strains (9.1%). Several studies have emphasized that *C*. *coli* isolates are more likely to acquire resistance than *C*. *jejuni* isolates [25], [26]. The differences in resistance between human and poultry strains may indicate that broilers are not the only source of *Campylobacter* infection in our population.

Mutation in codon 86 from ACA to ATA in the *gyr*A of *C*. *jejuni* and from ACT to ATT in the *gyr*A of *C*. *coli* has been reported to be the main mechanism of ciprofloxacin resistance. It has been shown that factors other than mutations in the QRDR of *gyr*A, such as in efflux pump gene expression, may contribute to phenotypic resistance [27]. In this study, DNA sequencing demonstrated direct correlation between the molecular tool for detection of a point mutation at position Thr-86 in the *gyr*A gene product and the MICs of ciprofloxacin, norfloxacin, and nalidixic acid. This result was similar to the one shown by Alonso *et al*. [12] and El-Adawy *et al*. [28]. The high prevalence of quinolone resistance could be related to the introduction of fluoroquinolones in the poultry industry [29].

Concerns about the development of resistant bacteria as a consequence of antimicrobial use in animals and the possible transfer of resistant strains from products of animal origin to humans have led to global changes in antimicrobial use in animal production systems. [30]. Although DNA sequencing is the most accurate technique for the detection of nucleotide mutations, this method is impractical to use as a routine diagnostic tool in many laboratories because the protocols are usually expensive and time-consuming [13]. Targeted molecular techniques offer an alternative means of assessing antimicrobial resistance among bacterial isolates [13], [21] and PCR-RFLP is a simple method for the detection of resistance in *Campylobacter* spp., particularly because the mutation appears to be always expressed phenotypically. The findings in this study contribute to our understanding of fluoroquinolone resistance of human and poultry *Campylobacter* spp in Brazil and emphasize the need for restricted use of antimicrobial agents in food animals to prevent resistance and ensure their use for treatment.

## Supporting information

S1 Table Minimal inhibitory concentration (MIC) analysis of 52 *Campylobacter jejuni* and *Campylobacter coli* strains isolated from poultry and human samples. (XLSX)

S2 Table Interpretation of minimal inhibitory concentration (MIC) analysis of 52 *Campylobacter jejuni* and *Campylobacter coli* strains isolated from poultry and human samples. (XLSX)
